# Qualitative assessment of community health workers’ perspective on their motivation in community-based primary health care in rural Malawi

**DOI:** 10.1186/s12913-022-07558-6

**Published:** 2022-02-11

**Authors:** Myness Kasanda Ndambo, Fabien Munyaneza, Moses Banda Aron, Basimenye Nhlema, Emilia Connolly

**Affiliations:** 1 Partners In Health/Abwenzi Pa Za Umoyo, PO Box 56, Neno, Blantyre, Malawi; 2 Division of Pediatrics, University of Cincinnati College of Medicine, 3230 Eden Ave, Cincinnati, OH 45267 USA; 3 Division of Hospital Medicine, Cincinnati Children’s Hospital Medical Center, 3333 Burnet Ave, Cincinnati, OH 45529 USA

**Keywords:** Motivation, Community health worker (CHW), Primary health care, Community-based health care

## Abstract

**Background:**

Community Health Workers (CHWs) have a positive impact on the provision of community-based primary health care through screening, treatment, referral, psychosocial support, and accompaniment. With a broad scope of work, CHW programs must balance the breadth and depth of tasks to maintain CHW motivation for high-quality care delivery. Few studies have described the CHW perspective on intrinsic and extrinsic motivation to enhance their programmatic activities.

**Methods:**

We utilized an exploratory qualitative study design with CHWs employed in the household model in Neno District, Malawi, to explore their perspectives on intrinsic and extrinsic motivators and dissatisfiers in their work. Data was collected in 8 focus group discussions with 90 CHWs in October 2018 and March–April 2019 in seven purposively selected catchment areas. All interviews were audiotaped, transcribed verbatim, coded, and analyzed using Dedoose.

**Results:**

Themes of complex intrinsic and extrinsic factors were generated from the perspectives of the CHWs in the focus group discussions. Study results indicate that enabling factors are primarily intrinsic factors such as positive patient outcomes, community respect, and recognition by the formal health care system but can lead to the challenge of increased scope and workload. Extrinsic factors can provide challenges, including an increased scope and workload from original expectations, lack of resources to utilize in their work, and rugged geography. However, a positive work environment through supportive relationships between CHWs and supervisors enables the CHWs.

**Conclusion:**

This study demonstrated enabling factors and challenges for CHW performance from their perspective within the dual-factor theory. We can mitigate challenges through focused efforts to limit geographical distance, manage workload, and strengthen CHW support to reinforce their recognition and trust. Such programmatic emphasis can  focus on enhancing motivational factors found in this study to improve the CHWs’ experience in their role. The engagement of CHWs, the communities, and the formal health care system is critical to improving the care provided to the patients and communities, along with building supportive systems to recognize the work done by CHWs for the primary health care systems.

**Supplementary Information:**

The online version contains supplementary material available at 10.1186/s12913-022-07558-6.

## Background

Community Health Workers (CHWs) are recognized as an integral part of the primary health care (PHC) system essential for achieving Universal Health Coverage (UHC) [1, 2]. The World Health Organization (WHO) recognizes CHWs as lay health care workers who are members of the community where they are selected and are answerable to the same community they are working for while supported by the health system [3–5]. CHWs are a vital, diverse group that links people from their homes and communities to critical health and social services within their communities [6]. Their responsibilities may include screening, providing education, promoting access to and linkage to PHC services, engaging in highly specific disease-related activities such as medication observation and adherence, and even direct patient care [4, 7, 8]. Through these roles, CHWs have shown potential for improving population health by preventing and managing various chronic diseases in geographically and economically diverse settings in low and middle-income countries [4, 8–12].

With the broad scope of work, CHW programs must balance the breadth and depth of tasks to maintain high-quality care delivery and CHW motivation [13, 14]. Previous studies suggest that CHWs are motivated by trust and respect from patients and the community, managers’ support and encouragement, and health knowledge acquisition that can be shared in their communities. They are dissatisfied by poor incentives and working conditions, limited health supplies and equipment, disrespect and limited support from the formal health care workers, and lack of transportation [15–18]. These motivation factors are broadly reported, but the contextualization is less commonly described, especially in a rural household-based CHW programs to inform program implementation and improvement.

This study uses the Herzberg’s dual-factor theory [19] of motivation to describe factors from the CHW perspective that stimulate or decrease motivation among CHWs in rural Malawi to inform programmatic optimization. The motivation-hygiene theory postulates that distinct factors cause job satisfaction and motivate workers with different factors causing dissatisfaction [19]. Factors that motivate workers are intrinsic to the work itself and include achievement, responsibility, trust, leadership, opportunities for advancement, recognition, and the work itself. Extrinsic factors -independent from the actual work - cause dissatisfaction in work and are linked to incentives, working conditions, resources, quality of leadership, and job security. Intrinsic factors will increase motivation and productivity, but extrinsic factors can reduce motivation if absent. Prior studies have successfully described intrinsic and extrinsic factors for CHWs but have primarily focused on challenges [15, 20–22].

Understanding intrinsic and extrinsic factors in community health worker programs can help structure programs for improving performance, health worker motivation, and retention to inform improvements in implementation [23, 24]. To explore CHW’s perspectives on their job satisfaction, motivation, and challenges affecting their performance, we conducted a qualitative study utilizing focus group discussions with CHWs in rural Malawi. The knowledge on factors from the CHW perspective that impact performance and thus program effectiveness will inform CHW leadership on programmatic and motivation adjustments to maximize care.

## Methods

### Setting

Neno district is a remote, rural, and impoverished district situated in Southwest Malawi with an estimated 144,442 people in 2020 [25]. The district hospital is not accessible by tarmac roads due to mountainous terrain and poor infrastructure. The majority of the population of Neno are subsistence farmers who live on less than 1.90 USD per day, and 95.5% do not have access to electricity [26].

### Community health worker household model in Neno District

At the inception of the CHW program in 2007, CHWs were positioned to support and accompany TB and HIV patients on clinical visits, treatment adherence, and psychosocial support that led to improved patient adherence and clinical outcomes [27, 28]. In 2016, the CHW program transitioned to the polyvalent household model. In this model, each household in the district is assigned a CHW regardless of disease for universal coverage. The household model focuses on eight major disease areas: 1) TB; 2) HIV; 3) STIs; 4) non-communicable diseases (NCDs); 5) family planning; 6) maternal and neonatal health; 7) child health, and 8) malnutrition screening in children under 5 years old [29].

Structurally, the household model is a three-tiered program with CHW, senior CHW (SCHW), and site supervisor (SS) roles designed in alignment with the national community health worker structure. Community health workers are called Health Surveillance Assistants (HSAs). HSAs are part of the Ministry of Health (MoH) Environmental Health Department. They are one of the most prominent cadres of the Ministry of Health workforce across Malawi, with over 9400 HSAs in service [30]. They support Village Clinics for preventive care for under-five children and pregnant women and manage care for pediatric malnutrition and tuberculosis cases [30, 31]. At the community level in Neno, one HSA serves approximately 540 households with case-finding, prevention, and essential case management. In terms of individuals, this translates to approximately a ratio of 1 HSA to 2300 people, which leads to poor coverage where households may go for long periods without interacting with their assigned HSAs [30, 32]. Thus, the household model was designed to complement the HSA program with a large cadre of > 1000 CHWs to act as foot soldiers to the HSAs. The HSAs frequently interact with SS to identify clients to follow up and other tasks for the CHW and SCHW and coordinate on village clinics and activities.

The Neno district population has 14 catchment areas, each served by a health facility - 12 primary health facilities and 2 hospitals - Neno District Hospital and Lisungwi Community Hospital. The household model recruits CHWs by catchment area with mapping to determine the overall number of households and the required CHWs to serve the community adequately. Then through engagement meetings with community leaders and structures, potential CHW candidates are nominated based on a designated selection criterion. Selected CHWs are assessed for literacy, and successful candidates are further taken through a five-day foundational training with subsequent quarterly one-day refresher training. Working materials are provided in the form of job cards, registers, and logbooks. Supervisory structures of senior CHWs and SSs receive further training and meet regularly with CHWs for ongoing supportive supervision and mentorship.

#### Community health worker role description

The primary roles of the CHW are to monitor the health of their assigned households, conduct health education activities, screen and link community members to essential health services at the health facility, collect data for reporting, and support patients and community members in their assigned households. The CHW’s roles are summarized into five main categories, namely; 1) monitor/screen; 2) educate; 3) collect data and report; 4) accompany/refer; and 5) support. Each CHW is assigned 20–40 households within their community, depending on village size and geography. They are expected to visit each household at least once a month with frequent visits to households with members who have active disease or need of follow-up or support. CHWs receive a monthly stipend of approximately USD 22. CHWs are supervised by senior CHWs and SSs within the 14 catchment areas in the district who work closely with the facility-based MoH community health care workers – the health surveillance assistants (HSAs). They meet regularly in a month at the community and facility level to review and validate data, receive program updates and plan their work.

#### Senior community health worker role description

SCHWs work as CHWs attending to ~ 15 assigned households as above and have an advising and supervisory role. They support village-level monitoring and supervision of 10–15 CHWs with verifying data and troubleshooting household challenges—the SCHWs complete spot checks with mentorship and coaching with the assigned CHWs each quarter. Additionally, the SCHW serves as a community TB sputum collection agent, where sputum is collected from presumptive TB clients and submitted to the health facility. They receive a monthly stipend of approximately USD 33 and are supervised by the SS with quarterly visits at the household level and participate in the monthly data reviews.

#### Site supervisor role description

SSs serve as the primary link between the CHW program management and MoH’s facility-based community health team. SSs supervise and mentor SCHWs and CHWs through spot checks, data monitoring support, and supervisory meetings. They are responsible for data aggregation, record keeping, and monthly reporting. They jointly work with the HSAs to ensure CHW collaboration with specific emphasis on TB and malnutrition programs.

### Study design

We utilized an exploratory qualitative design of focus group discussions (FGDs) with CHWs and SCHWs to examine CHW perspectives on facilitating factors and challenges on their ability to perform their duties. FGDs were conducted in October 2018 and March–April 2019. Of the 14 catchment areas in the Neno district, seven catchment areas (Midzemba, Zalewa, Chifunga, Ligowe, Neno District Hospital, Nsambe, and Dambe) were purposively selected to take part in this study per topography. We purposively selected ninety (90) participants from these catchment areas with consideration of four criteria; 1) the type of CHW (CHW versus SCHW), 2) CHW age to include both young and old participants, 3) gender to include a balance of males and females, and 4) duration of work as CHWs (“new” to include those working < 5 years; “old” to include those working > 5 years) (Table 1). We chose this approach to recruit a diverse group of CHWs and SCHWs with different experiences to find diverse perspectives on their enabling factors and challenges that affect their performance.

**Table 1:** Demographic characteristics of CHW and SCHW focus group participants

Variable	N (%)
Age	
25–35 years	33 (36.7)
36–46 years	39 (43.3)
47–57 years	17 (18.9)
58–68 years	1 (1.1)
Gender	
Male	41 (45.6)
Female	49 (54.4)
Role	
CHWs	31 (34.4)
Senior CHWs	59 (65.6)
Duration^a^	
New CHW/SCHW	31 (34.4)
Old CHW/SCHW	59 (65.6)

^a^New = < 5 years working; Old = > 5 years working

### Data collection

In October 2018, four FGDs of CHWs and SCHWs were conducted. Each FGD comprised 9–12 participants, with one FGD in the catchment of Zalewa, Chifunga, Ligowe, and Dambe. In March – April 2019, an additional four FGDs were conducted with SCHWs only with 9–12 participants per group with one FGD in the catchments of Midzemba, Chifunga, Neno District Hospital, and Nsambe.

The study team developed two question guides for this study (Appendix 1 and 2). One question guide was developed for the combined FGDs (CHWs and SCHWs), and the second question guide targeted SCHWs with a focus on their supervisory roles with overlapping question prompts. For the combined FGDs (CHWs and SCHWs), we asked participants on knowledge and perspective of CHW services, CHW’s influence on health care seeking and community trust, inter-program relationships, perceived differences between CHWs and other health cadres such as HSAs, and ideas for program improvement.

The guides were developed in English, translated into the local language of Chichewa, and pretested with CHWs and SCHWs in the Neno District Hospital catchment area before use. We did not recruit CHWs and SCHWs who participated in pretesting for the main study. The content and translations were adjusted from feedback before formal data collection. The organization recruited the research fellow (MKN) and three research assistants for the evaluation study, with the research fellow facilitating data collection. These positions were not part of the implementation team and did not have prior knowledge of the household model in Neno, Malawi. The facilitator explained the study to participants and obtained signed written informed consent. The study was conducted by the Declaration of Helsinki guidelines and regulations [33]. Each FGD was recorded and took approximately 2 hours.

### Data analysis

Transcripts were transcribed verbatim in Chichewa and then translated to English and later uploaded in Dedoose version 8.3.17 for data management. Transcription was done by the research fellow (MKN) and double-checked by (BN), who listened to all audio recordings and verified the translation from Chichewa to English before loading them in Dedoose. The data were analyzed using qualitative content analysis [34]. The research fellow familiarized with the data set through immersion by the repeated and active reading of transcripts [35]. To ensure the reliability of coding and consistency, the research fellow, BN and EC independently read the first three transcripts line by line to deductively assign codes to similar concepts that repeatedly emerged from the data in line with study objectives [36]. The first codebook was generated from the first three transcripts through a consensus process by looking at commonalities and differences [36]. The research fellow then coded the rest of the transcripts, with feedback from the senior authors, deleted repeated codes, and added new ones until a final codebook was created. The final codebook was agreed upon by the joint consensus of all authors [36, 37]. We identified relationships between these codes, repeatedly identified codes were merged, and themes and sub-themes were generated from these codes. We chose quotes for each theme and sub-theme summarizing the main points [35, 36].

## Results

**Fig. 1:**
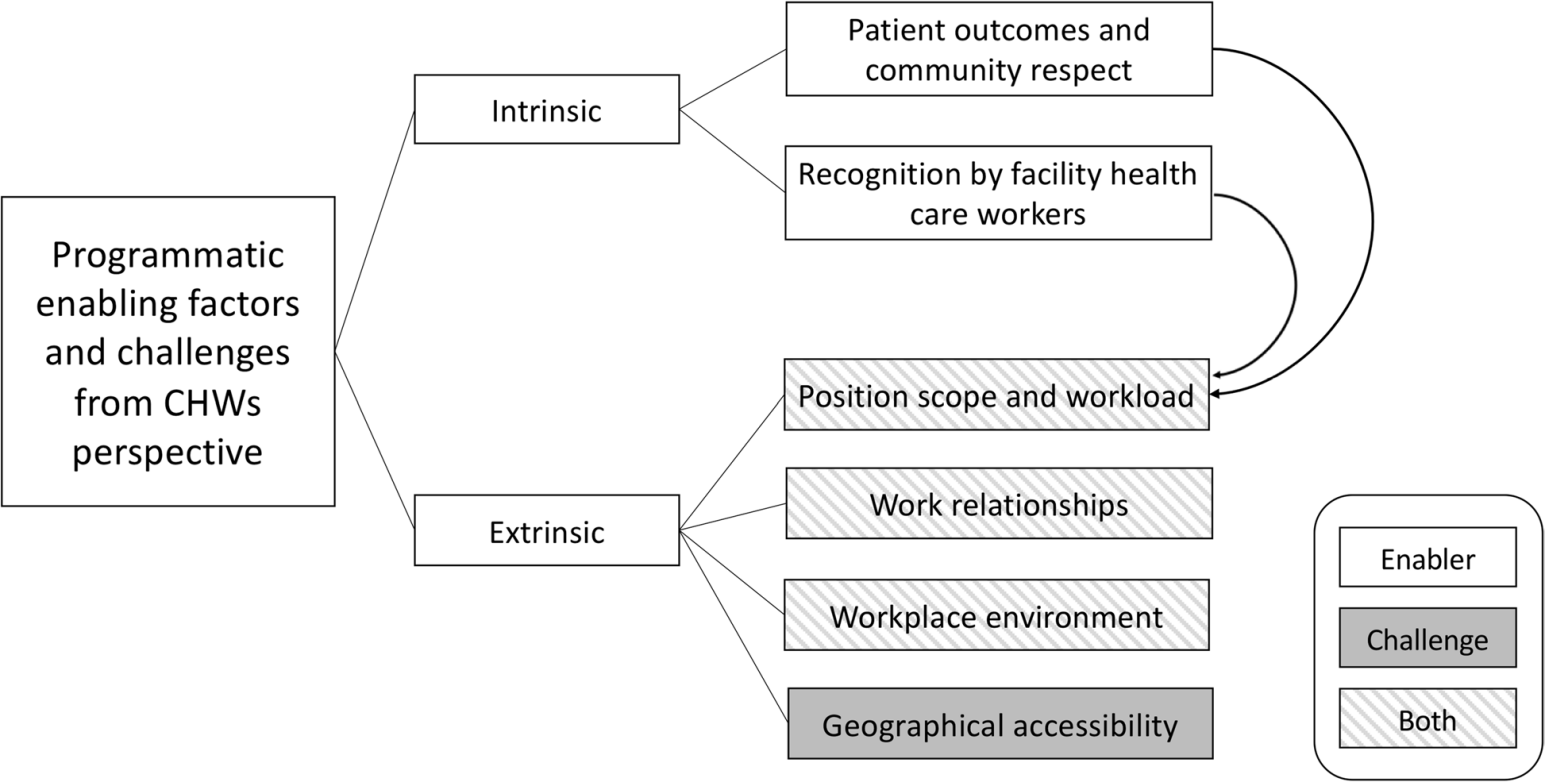
Mapping Enabling Factors and Challenges from the Community Health Worker Perspective in Rural Malawi

CHWs’ motivation and performance were influenced by diverse elements that arise from the complex context in which they work as a conduit between the community and the formal health care system. As the narratives of the CHWs were examined, findings were deductively identified around common themes of intrinsic and extrinsic enabling factors and challenges CHWs face in performing their roles. Intrinsic factors included patient outcomes, community respect and recognition by formal health care workers, which enabled the CHW performance, but led to a challenging extrinsic factor of increased workload. Extrinsic factors included position scope and workload, work relationships, workplace environment, and geographical accessibility. Most of the extrinsic factors had motivational aspects and challenges for CHWs except for geographical accessibility, which was a consistent barrier in this environment (Fig. 1).

### Intrinsic factors

#### Patient outcomes and community respect

Through the focus group discussions, it was clear that CHWs recognize they are valued and appreciated in their communities by providing support and care trusted by individuals. The CHWs reported they are considered ‘doctors’ and respected leaders in rural communities where most families do not have immediate access to facility-based healthcare. Through this confidence in the CHW work, individuals were able to seek care at facilities with positive results and even sometimes life-saving treatment, which built gratitude for the CHW with social connectedness between the community and the CHWs. The CHWs were motivated by this relationship to enhance their performance, and it fostered professional status and trust in the CHWs.

*“One man was very sick and the relatives were thinking that he has been bewitched. When I suggested to take him to the hospital, they told me not to trouble myself because the man will soon die. I took the man to the hospital on my bike. He was tested HIV positive and I have been helping him to adhere to medication. Within two months, the man picked up. The relatives came to thank me that without me their relative would have died. Even himself he is very thankful to me. They see me as their king and I walk majestically because of that.”* CHW in Chifunga

*“Household model is good. In my area, I am able to differentiate how life was in the past and now. Before, we could find a lot of sick people in the homes due to long distances to the hospitals as well as poor health seeking behavior. People opted for traditional medicine. Now, having taught them through home visits, they rush to the hospital when sick and are careful on preventive measures.”* SCHW in Neno district hospital

With dependable, high-quality work and positive results from seeking care, CHWs reported that the community had great confidence in their abilities. Through the trust in the CHW abilities and training they receive, households and individuals would request CHWs to provide psychosocial support and advice with problems even beyond the supported health care topics such as marital affairs. One CHW shared;

*“ … we even help in marriage issues when there are some disagreements. We help resolve their disputes. When it is beyond us when the tension is too much, we refer them to social welfare … .”* CHW in Zalewa

CHWs reported embracing a unique supportive role in their communities beyond their expected duties. CHWs often stated spending more time than required per their job descriptions and often could even monetarily assist clients with hiring transport or helping with hospital-associated costs. These personal tasks contributed positively to clients’ well-being and instilled a relationship of gratitude with the CHW, which motivated the CHWs in their performance. However, this practice could lead to CHWs working beyond their routine tasks which may be a challenge in scope and resultant workload for the CHWs.

*“One of my households did not have a toilet and the man is weak so I personally dug the pit on his behalf.”* CHW in Dambe

*“I once helped a person who did not have relatives to support him at the hospital. He had TB, and I was his guardian … I also hired a motorbike to take the client to the hospital as he couldn’t have done it on his own.”* SCHW in Ligowe

#### Recognition by facility health care workers

Participants reported an excellent working relationship with formal health care providers with positive recognition of their work in the community. In the FGDs, CHWs reported that both cadres trust each other’s care of the clients. The CHWs stated that their work has improved the community’s health and decreased the number of clients requiring to visit the health facility. The CHWs further noted that the lower numbers at the facility, in turn, helps the health facility staff to lessen their workload and makes the CHWs feel proud of the care they are giving individuals. The recognition both by health facility staff and the clients with seeing the beneficial outcomes increased CHW confidence and motivation in their performance.

*“When I take a client to the hospital, doctors provide good care hence people in the community trust me more when they see positive outcomes after seeing the doctor... there is a good relationship with people who work at the hospital and people trust us when we ask them to go to the hospital that they will be healed.”* SCHW in Neno district hospital

*“Yes, we feel like we are trustworthy. Even healthcare providers at the hospital are able to note that with our coming in, their work pressure is manageable. If you go to the OPD [outpatient department], you will find that there are fewer people as compared to the queues that were there previously. All this is happening because we help people in the villages through home visits.”* SCHW in Midzemba

The CHWs reported acting as foot soldiers for the primary health care system at the facility level. With the CHW’s ability to support community primary health care, the over-worked HSAs utilized the CHWs to follow up on household-level tasks in their communities.

*“You know as CHWs we do the job that was previously done by HSAs. They would previously walk around the communities following up pregnant women and distributing chlorine. That is now done by us and they just get reports … so yes, they are happy with what we are doing in the communities”.* CHW in Zalewa

*“We work a lot. We have taken the place of HSAs as previously they could go around the community to monitor pregnant women but now, they no longer do that … we are the ones doing it. We do even more than what they were doing … we just give them reports.”* CHW in Dambe

### Extrinsic factors

#### Position scope and workload

Each CHW cadre (CHW, SCHW, and SS) has a specific job description and expectations with tasks aligned to support the eight disease focus areas in the household model. As demonstrated by CHWs in the FGDs, they have built trust and social connectedness in the communities they serve and with health facility staff by executing these tasks capably and efficiently. Even though this has motivated CHWs, it led to additional requests and demands on their time from clients and the health facility staff. CHWs perceived that their roles expanding past their original job descriptions challenged their knowledge and performance and made them feel under-appreciated. The increased scope was demonstrated through examples of where CHWs were asked to do tasks or counsel on disease areas they had not been trained on, or clients requested specific medications or tests that they do not routinely administer.

*“...We also add some stuff that we were not trained on such as malaria and sanitation. Sometimes even cancer we tackle. When they ask us difficult questions, we refer them to the doctors. So we already have too much work.”* CHW in Dambe

*“Clients ask us for simple supplies like paracetamol and some contraceptives. Sometimes people walk long distances to the hospital just to receive pain killers. The community expects us to have such in stock for easy accessibility.”* CHW in Chifunga

Similarly, at a health facility level, CHW participants described that their ability to reach clients at the household level and provide high-quality screening, referral, and care placed their services in high demand. Therefore, when designing new programs, the organization or health facility included new tasks requiring CHW involvement with expansion of care delivery.

*“ … Also there are some projects that just come in and require our involvement since we are best suited in the communities … .”* SCHW in Midzemba

Again, this recognition and value in CHW work enabled their performance. However, even though this shows the highly valued work the CHWs perform, with too many additional tasks and increased scope, it increased CHW workload and scope which challenged their performance.

*“ … And then they keep adding jobs … because we have a lot of tasks. After 3 weeks we are supposed to submit reports to our supervisor so we really work under pressure … ”* CHW in Zalewa

In the household model, the number of households in original job descriptions was set to 20–40 for CHWs and 10–15 for SCHWs. However, with adjustments in household assignments due to village growth, replacement of CHWs, and sometimes personal relationships with clients, many of the CHWs reported taking on additional households above the target number. CHWs indicated that this could be caused by their own motivation to take on additional households. However, it could also be due to attrition, geographical adjustments, or other CHW’s lack of work in other circumstances. No matter the reason, with more households, the CHWs had an increased workload, which affected their performance.

*“The number is too big. 40 is just a limit as for me I have 54 houses. I cannot work efficiently as a volunteer. I am just a volunteer as such I fail to visit all the houses in one month as a result some are not visited.”* CHW in Ligowe

*“I was given 15 households but now I have 25 as some households were not comfortable with their CHWs and some CHWs had more households and I just had to relieve them. As such, I have more pressure and my output is affected.”* SCHW in Midzemba

### Workplace relationships

There is a supervision structure in the household model as described in the methods with CHWs reporting to SCHWs and SCHWs reporting to SSs. CHWs reported that this structure with mentorship and supportive supervision facilitated relationships between CHWs and the overall function of the program. Having two higher-level cadres to support CHWs, allowed for knowledge and skills transfer, improved delivery of tasks and shared supervisor and peer support in working through challenges. This support enabled the CHWs to perform their tasks to the high standards of care.

*“When I have a problem, I make it known to the senior so that I can be assisted before the issue goes to the site supervisor... The Site Supervisor also helps us with skills on how to approach the household which may be difficult or unapproachable. They also visit our households. They teach and then tell me where I have done well as well as where I have not done well and then corrects me.”* CHW in Zalewa

*“We interact well with our CHWs. We hold meetings where we discuss challenges that they meet so that together we can find a solution. The frequent meetings help us to build a good relationship and work together. We also mentor them in areas where we feel that they are not doing well. We interact with the Site supervisor during the meeting when we submit reports and we discuss issues that we have failed to handle in the community.”* SCHW in Dambe

Despite many positive outputs through the supervisory and peer support structure, there were some difficulties reported in interpersonal relationships between CHWs that challenged performance. This was discussed in the FGDs most often through the example of a CHW who is older or has worked longer than their supervising SCHW. In some situations, these differences led to the older or more experienced CHW not being open to constructive criticism or instructions from the supervising SCHW, leading to a strained interpersonal relationship on both sides. However, it was discussed that site supervisors had aided in building positive working relationships between CHWs and SCHWs, which had improved this challenge.

*“Some CHWs who have worked longer than us the SCHWs do not take instructions as they think they know the work better than we do. Of course, the SS has been tackling this issue such that the current situation is better off than at first.”* SCHW in Midzemba

### Work environment

The household model provides CHWs with teaching aids such as charts, job cards, and booklets used for education in households. With these inputs, CHWs felt enabled to complete tasks and are easily identified in the community as health care workers as they carry the materials from house to house.

*“At some point we were given bags so when we move around carrying our registers in those bags, we could hear people pointing at us that those are health workers. It feels good to be recognized as somebody in the community. Some even call us doctors when they see us with our teaching aids. So, by giving us such supplies, we feel very good.”* CHW in Zalewa

Despite the educational materials enabling the CHW work, CHWs reported the need for additional and enhanced teaching aids to continue their high-quality services. The CHWs expressed that with updated educational materials or teaching aids to leave with clients, would have an increased beneficial impact on the community.

*“Here in Ligowe we do not have enough teaching aids. We would therefore appreciate if we can be considered e.g. family planning, some have, others do not have … enlarge font for teaching aids as some elderly clients fail to see properly … we should have more teaching aids so that you leave them a copy to go through and then ask questions during the next visit. Some people even ask us to be writing notes for them so that they can have notes to go through when we are away.”* CHW in Chifunga

Additionally, the CHWs work in very rural and remote terrain with several months of daily rainfall that challenges their ability to deliver services. CHWs expressed that regularly replenished protective gear would allow them to more frequently visit all assigned households and motivate them through the show of support with these inputs.

*“As we approach the rainy season, the management should consider buying us necessary materials e.g. boots, raincoats and necessary bags. We feel complete with such supplies … it has taken long since we were given the same and they are worn out.”* CHW in Dambe

Even though attrition overall is low in the household model, with a workforce of > 1200 members, it occurs that CHWs need to be replaced. CHWs reported a challenge when new CHWs are recruited within the household model and are not immediately trained by the CHW leadership. Then existing SCHWs are required to visit households without a current CHW, and then when the replacement is hired, sometimes even train him or her. Participants noted that when this occurs, the added work on the current CHW results in demotivation. Furthermore, there were concerns by the CHWs that the replacements trained only by the lower-level cadres might not be best prepared for scheduled tasks and performance to the standards set in the household model.

*“Yes, though not a lot but we have CHWs who resigned and opted for greener pastures … on top of our heavy workload, we have to provide on job training for the new joiners. Mind you, such CHWs cannot perform the same way as those that went through normal training since we can also forget some things. It is important that management should look into this seriously.”* SCHW in Midzemba

In the household model, CHWs and SCHWs are hired as volunteers who receive a stipend for their work with the expectation that it supplements other income or work. However, during FGDs, the CHWs noted that the routine tasks in the expected time were too much for a volunteer and challenged their performance. CHWs expressed that their stipend should increase or be fully employed by the organization, which would further recognize them as trusted primary health care professionals; and, in turn, motivate them.

*“The work is too much such that we do not find time to do our own things that can help us generate income to earn a living, we would therefore appreciate if our stipend is increased so that we do not complain if we spend more time on it.”* CHW in Zalewa

*“As you know we are volunteers. Much as we appreciate the money we receive, in real sense it is too little, so we need to be doing other things to earn a living. If they can hire us as employees, I think it could be good for us as we will be able to dedicate our time to the fullest. In this way, things will go on smoothly*.*”* CHW in Ligowe

### Geographic accessibility

Neno district has very challenging and mountainous terrain, especially in the rainy season with flooded rivers, mudslides, and impassable roads. Furthermore, some villages and areas have households with long distances in between them. Thus CHWs reported mobility challenges even with reasonable household allocations, which challenges their performance. There were recommendations for transport support to improve outputs.

“Some of us *walk long distance to reach households that are within our villages so they should provide us with bicycles. In my case, sometimes I work throughout the day just to cover three households because my households are very far apart...”* CHW in Zalewa

Due to a broader geographical area in supervision and sputum collection, SCHWs are provided transport support with bicycles. However, SCHWs reported that with rough conditions and a lack of repair, the bicycles break down and are unusable. Similarly, to no transport support, the lack of reliable transport decreases the SCHW performance. There were appeals made to improve transport conditions for enhanced performance for all CHWs.

*“I travel long and hilly distances to the facility hence the bicycles that we have do not help at all … at least if we had push bikes … sometimes they break down on our way to the hospital to submit sputum and we just return back home despite covering a long distance. This demotivates us.”* SCHW in Dambe

## Discussion

By utilizing the dual-factor theory, examination of intrinsic and extrinsic factors can enhance understanding of enabling and challenging factors in CHW performance to improve primary healthcare delivery. The results of this exploratory study of CHW show that enabling factors are mostly intrinsic with positive patient outcomes, community respect, and recognition by the formal health care system. Extrinsic factors can also enable CHWs to work in supportive relationships between CHWs and supervisors and provided educational materials to enhance performance. The challenges mainly lay within extrinsic factors: increased scope and workload, lack of up-to-date resources and transport support, and rugged geography. One interesting connection between intrinsic and extrinsic factors was that the CHWs were motivated by intrinsic factors of positive patient outcomes and the corresponding trust and confidence in their care by the community and health facility staff through their capable work. However, this led in some circumstances to extrinsic challenges of additional workload and increased scope of work that led to CHW performance and demotivation challenges.

Most of the intrinsic factors in the household model were enabling from the perspective of the CHWs. Through the CHWs providing high-quality screening, referral, and patient follow-up, the patients have improved health knowledge, care-seeking behaviors, and outcomes. Furthermore, the CHWs felt well respected and trusted in their communities, with relationships built on trust and empathy from this beneficial impact. This echoes findings in other studies where CHW’s motivation stems from interpersonal relationships of trust and social standing in the community [38–42]. These factors encourage CHWs to improve performance and extend themselves to help their communities, leading to deeper relationships with individuals and going above and beyond their job description. Examples in this study include paying out of pocket for patients’ healthcare costs, providing housework, or moderating interpersonal relationships. Even though these actions were altruistic and born from the firm social connections between the CHWs and their patients and communities, it challenges the CHWs in expanding the scope and work required beyond their formal expectations. Similarly, Oliver et al. [14] demonstrated that the integrative role of CHWs enables effective care and places CHWs straddling the tasks of formal health care providers and more of a “social work” function within the community that can lead to tension and dissatisfaction.

Participants reported an excellent working relationship with the formal health care providers with the intrinsic factor of recognition of their work which motivated the CHWs. A Cochrane review [38] on barriers and facilitators of CHW implementation showed that support from health systems gives CHWs creditability. Furthermore, health professionals often appreciate the CHW’s contributions in reducing workload along with their communication skills, referrals, and commitment to patients [38].

However, one challenging extrinsic factor is that with increased CHW scope and workload imposed by themselves and health facility staff, CHWs can feel demotivated with barriers to optimized service delivery. For example, the CHWs often meet additional requests for information and screening on tasks outside the eight disease focus areas, expanded scope of responsibilities, and take on additional households. Task shifting is typical in CHW programs in low-resourced settings but is an important extrinsic factor that can lead to demotivation and decrease program effectiveness [1, 38, 43–45]. In Malawi specifically, a prior study conducted on the community health workers at the facility level (e.g., HSAs) demonstrated that heavy workload and competing tasks lead to health care workers not performing all their assigned tasks [46]. Therefore, it is not surprising in this context that health facility staff may utilize the CHWs to perform some of their tasks due to their own overburdening. In this sense, the use of CHWs as “foot soldiers” for the HSAs is successful task shifting as seen in other settings and endorsed by the World Health Organization (WHO) to tackle health worker shortages [47–49]. However, performance and motivation can waiver with task shifting, as suggested by the CHWs in this study and others [47, 50]. As clearly defined by the WHO, checks, and balances on workload and provider capabilities are required to protect the health care workers and patients in care from maintaining high-quality care and performance [47]. Therefore, to successfully utilize the HSAs and CHWs to provide care in the rural Neno district, we must ensure both cadres have clarity of responsibilities with enough staff, time, and training to perform tasks adequately. They must be supported with solid leadership and governance without overburdening with resultant demotivation and potentially poor performance with poor client care.

Challenging extrinsic factors included workplace environment, training and resources, and difficult geographical accessibility with lack of transportation. Other CHW studies in India and South Africa highlight similar barriers [16, 22]. Training and capacity building are vital components of CHW programs as it distinguishes CHWs from being just another concerned member of the community. However, we found that training sometimes was left to the CHW supportive supervision structure, which added to their workload and dissatisfaction. Furthermore, there was concern that training by peers could be substandard to formalized training by CHW leadership. Out of date or inadequate job aids, equipment, and resources also constrain CHW effectiveness and motivation. Without all relevant resources and tools, CHWs feel that they are not giving the health education desired by the community and may become less relevant. Even though monetary incentives are debated within CHW programs, we found that CHWs appreciated their stipend in exchange for their professional services. Furthermore, due to the tasks at hand, time requirements, and needs within the community, through the FGDs the CHWs requested increased stipends or full employment benefits from the organization to match the amount of work expected and to motivate them. Similarly, other community health worker studies in Malawi and beyond have identified low salaries as dissatisfiers to their work [32, 38, 44, 50].

In community-based care, challenging geographical environments and inadequate transportation are common problems, especially in mountainous and poorly developed areas [1, 9, 51, 52]. Although the program was designed for CHWs to provide services within their community and within walking distance, the reality from the discussions with CHWs is that they can cover much larger areas. Without reliable transportation and added households outside their communities, the lack of accessibility added to CHW workload and travel decreased performance and household visits. Likewise, other studies have shown that frequently required visits, broad geographical coverage, and lack of transportation were dissatisfiers of the CHW work environment [22, 50, 52]. Considerations for programmatic improvement include redistribution of households to decrease walking distances and limit household numbers and investigation of reliable transportation solutions.

Our findings show that bidirectional solid and supportive relationships between CHWs themselves and supervisors encourage collaborative work and increase morale. Barriers to interpersonal relationships between CHWs can be due to older CHWs or more experience reporting to those younger or without experience. This interpersonal relationship factor is extrinsic through the relationships and work environment but has the intrinsic aspect of peer recognition. Similar to our findings, prior studies on CHW performance show that supportive supervision and mentorship are critical to program success, along with the opportunity to share experiences with fellow CHWs [11, 15]. Programmatic improvement considerations include clear, high-quality performance standards for supervisors not based on age or length of service with clear communication to all CHWs with work on productive and bidirectional relationships between CHWs and supervisors.

CHWs are critical “bridges” between the community and the facility-based primary health care system that provide competent care within the community [4]. This study demonstrated important enabling intrinsic and extrinsic factors for CHWs such as education and teaching aids, robust supportive supervision, positive patient outcomes, and trusting relationships between CHWs and communities and health facility staff. However, there is also a need to improve both levels to facilitate CHW motivation and performance with key recommendations from this study. Firstly, we must ensure CHWs and health facility staff, including HSAs, have clarity of responsibilities with adequate staff, time, inputs, and training to perform tasks adequately. Secondly, strong leadership and governance are required to ensure positive client care and outcomes that motivate health care staff with optimized performance.

Furthermore, attention to interpersonal relationships between peers and supervisors must be prioritized with adequate training, teaching resources, transport, and monetary incentives to motivate high-quality work. Lastly, many CHWs are deployed in rural and remote areas taking care of clients without immediate access to facilities. We must ensure that CHW leadership structures and CHWs jointly set scheduled tasks, scope, and geographical spread for a motivated and productive CHW workforce to provide high-quality care delivery to rural populations. Further investigations into CHW performance may include health facility staff perspective and expectations of CHW work, valuable support systems for CHWs, optimized educational tools from the perspective of health facility staff, CHWs, and clients, how to facilitate CHW peer, community, and facility recognition, and trust.

### Limitations

This study was conducted in a small rural and remote district in Malawi, limiting generalizability. However, Neno District has similar geographical and socioeconomic aspects to many rural areas in Sub-Saharan Africa, with several themes shared amongst the literature on motivation and job performance in CHWs. The study was conducted in the stated household model program focusing on screening, linkage, and patient follow-up without CHWs providing direct diagnostics or medication, which differs in scope from other CHW programs even within Malawi and other countries. However, many of the challenges and enablers found in this study are universal for health care workers, as demonstrated in other studies referenced, and can be utilized when implementing or improving community health worker programs and projects.

## Conclusion

This study demonstrated enabling factors and challenges for CHW performance from their perspective within the dual-factor theory. We propose an enhancement of capable and motivated CHWs to facilitate trusting relationships that lead to positive patient outcomes while improving the clarity of responsibilities with adequate staff, transport, and training to perform tasks adequately. We recommend CHW programs provide robust training and education, supportive supervision, and leadership with jointly set scheduled tasks, scope, and geographical spread for a motivated and productive CHW workforce. Empowering CHWs by raising awareness and working together to mitigate challenges and enhance motivational factors can engage them as active partners in programmatic improvement to deliver high-quality primary care in the community.

## Supplementary Information

**Additional file 1.**

**Additional file 2.**

## Data Availability

The dataset generated and analyzed during the current study is not publicly available. Even without identifiers such as names, the dataset could potentially hold identifiable participant information in aggregate form due to sex and catchment area along with potential disease status. Neno District is a small district, and with potential identifiers, we believe it would be ethically inappropriate to publicly share the data that could reveal our participants’ identities if read by someone within the district. The dataset or part of it could be available from the corresponding author on reasonable request with permission from the Neno District Research Committee (apzuresearch@pih.org).

## Abbreviations

CHWs: Community Health Workers
PIH: Partners In Health
APZU: Abwenzi Pa Za Umoyo
TB: Tuberculosis
NCDs: Non Communicable Diseases
STIs: Sexually Transmitted Infections
FGDs: Focus Group Discussions
SCHWs: Senior Community Health Workers
SSs: Site Supervisors
HIV: Human Immunodeficiency Virus
ART: Antiretroviral Therapy
HSA: Health Surveillance Assistant
