# Supplementary material for: Qualitative assessment of community health workers’ perspective on their motivation in community-based primary health care in rural Malawi
Source: BMC Health Serv Res. 2022 Feb 11;22:179. doi: 10.1186/s12913-022-07558-6 (PMC8840069; doi:10.1186/s12913-022-07558-6)
Supplement: Supplementary file 1 — Additional file 1. [file 12913_2022_7558_MOESM1_ESM.docx]

**QUALITATIVE ASSESSMENT OF COMMUNITY HEALTH WORKERS’ PERSPECTIVE ON THEIR MOTIVATION IN COMMUNITY-BASED PRIMARY HEALTH CARE IN RURAL MALAWI**

**APPENDIX 1: CHW & SCHW Focus Group Question Guide**

| **Questions (& probes)** | **What Question Measures** |
| --- | --- |
| 1. What roles do CHWs play in the community?  - What are the ways that you think CHWs should support clients? - How would communities be different if there were no CHWs working? | “Icebreaker question”; also a good way to see whether CHWs independently mention anything relevant to social connectedness. |
| 1. Let’s talk about what a typical day of work looks like for you.  - Can you walk me through what a monthly household visit looks like? - Can you walk me through what a patient visit looks like?   - Of the activities mentioned, which ones take up the most time?   - Do you ever spend all day doing home visits? How many times did you do that this week?   - In a typical week, how many patients do you accompany to the health facility?   - How many minutes’ walk is the closest house from where you stay? The farthest house? | general understanding of how CHW work and how time is spent |
| 1. Overall, what parts of your job do you think are the most helpful and effective at improving the health of the households you support?    - - Which of your assigned tasks are you most comfortable doing and which ones are most difficult?    - Are there any ways that your responsibilities could be changed to give you more time doing the things that are most helpful and important to your clients or the community?      - If yes, what would these changes be? | CHW uses of time and possible changes to improve program |
| 1. What are the services that CHWs are supposed to provide through the Household Model? | Knowledge and beliefs about what CHWs provide |
| 1. As CHWs, you are trained to focus on the following key health priority areas (HIV, TB, maternal health including ANC, facility-based deliveries, post-natal care), under-5 malnutrition, STI and NCD)s.  - Do you think these are the best areas for you to focus on, or would you recommend any changes? | Acceptability of Focus areas |
| 1. Are CHW facilitating relationships beyond their scope of work with the community as well as household members?  - Have you been able to help patients find support in the community that they did not otherwise know about? - If so, can you give me a specific example? - For CHWs who worked within the old model: Since switching over to the new model where you work with a whole household, has your relationship with patients changed at all? If so, how? - What, if any, do you feel is a CHW’s effect on the larger community apart from the family they are assigned to? | Social connectedness |
| 1. What (if any) behavior changes have you seen over time in the households you work with?    - Can you tell me about a specific time when you helped a patient do something that they would not have done without your support? | CHW influence on health care seeking attitudes and behaviors |
| 1. In your role as CHWs, are you able to impact treatment adherence? How so, or why not?  - Provide examples from your encounters with clients/households. | Treatment Adherence and Default |
| 1. As a CHW, in what ways are you helping to address health conditions impacting your community?  - Can you think of an example when you or another CHW identified a person or household and linked them to the health facility when they would not have otherwise gone for care? | Linkage to Care |
| 1. What are your interactions with your supervisor like?    - Can you describe any helpful things your supervisor does for you?    - Can you think of any things your supervisor does that are less helpful or you wish might be different?    - What kind of support would you require for you to best perform on the most difficult tasks?    - Are there supports that CHW supervisors are not currently giving that you think would be helpful for you and other CHWs in the future? | Supervision and bi-directional communication |
| 1. Currently one CHW is assigned to 20-40 households. Based on your experience, is that a feasible number of households to give to one CHW? Why?  - What would be the highest number of houses a CHW could effectively support per day and why? - What is the most ideal number and why? | Ideal CHW to household ratios, solutions for scale-up |
| 1. Do you think community members feel equally comfortable with a male or a female CHW? Why/why not?  - How has gender impacted your experience as a CHW? - Do you think community members feel equally comfortable with a male or a female CHW? - Are there things that are best done by a female CHW but not a male? Or things that are best done by males, not females? - Do you think the gender of a CHW is important if they are supporting a pregnant mother? Or counseling around family planning? | Gender perceptions that may affect CHW program implementation |
| 1. As you probably know, recently APZU updated the CHW strategy to focus on households rather than individual patients.  - Overall, what has been your experience as a CHW now that APZU is using this household model for CHWs?   If you were a CHW before the household model came into place, what are the main differences in your work comparing now to the time before household model?  What do you like most about working within the household model?   - What are your main challenges with the household model? | General perceptions; interview wrap-up question |
