# Supplementary material for: Qualitative assessment of community health workers’ perspective on their motivation in community-based primary health care in rural Malawi
Source: BMC Health Serv Res. 2022 Feb 11;22:179. doi: 10.1186/s12913-022-07558-6 (PMC8840069; doi:10.1186/s12913-022-07558-6)
Supplement: Supplementary file 2 — Additional file 2. [file 12913_2022_7558_MOESM2_ESM.docx]

**QUALITATIVE ASSESSMENT OF COMMUNITY HEALTH WORKERS’ PERSPECTIVE ON THEIR MOTIVATION IN COMMUNITY-BASED PRIMARY HEALTH CARE IN RURAL MALAWI**

**APPENDIX 2: SENIOR COMMUNITY HEALTH WORKER’S FOCUS GROUP INTERVIEW GUIDE**

| **Questions and Probes** | **Themes** |
| --- | --- |
| 1. **As a SCHW, what tasks do you perform on a regular basis?** (*Allow them to come up with an exhaustive list of the tasks they do. You can probe them by asking which tasks they do at individual/patient level, household level, community level, facility level, any activity*)   - Of the highlighted tasks, which one takes most of your time doing? Why? - Do you think the most helpful things to the households are the ones that take up the largest amount of your time or is that not always true? Why? - SCHWs have three main responsibilities; to visit their assigned households on a monthly basis, to supervise fellow CHWs and to act as community sputum collection agents. If you were to rank these three in order of difficulty, which one would you rank first, second and third? Why? - Specifically, which elements are challenging about the highlighted area? - Is there anything you would like to change about what you do, so that you are more effective at your job? | “Icebreaker question”  **Tasks of SCHWs – Role Listing** |
| 2. **How many households do you have?**   - What is the highest number of households you are able to visit in a day? - How many minutes’ walk is the closest house from where you stay? The farthest house? - How many days in a week do you spend doing SCHW related tasks? - How many hours on average in a day do you spend doing SCHW related tasks? - In a typical week, how many days do you find yourself at the facility accompanying patients? - Are there instances at all where you are unable to conduct monthly home visits for all your assigned households? If yes, please highlight some of the reasons that hinder you from conducting all your monthly home visits? | **Time spent on Tasks/Distance** |
| 1. **What is your relationship like with your SS?**     - How often are you visited by your SS?    - On average, how much time do you spend with your SS when s/he visits?    - Could you describe what your SS does when s/he visits you?    - Do you find your SS’s instructions clear?    - What areas of your job do you find most challenging? Has your SS been able to help?    - Do you feel that you are adequately supported?    - Can you describe any helpful things that your SS does for you?    - Can you think of any things your supervisor does that are less helpful or you wish could be different?    - What kind of support would you require for you to best perform on job? | **Supervision - SS** |
| 1. **How many CHWs do you supervise?**  - How is your relationship like with your CHWs? - Based on your experience of supervising CHWs, which aspects of their jobs are they performing well on? Why - Which aspects are they struggling on? Why? - What aspects do you find the most difficult when supervising CHWs? - What support would you like to receive so that you are able to effectively supervise your CHWs? - Since you started work as a SCHW, how many CHWs have resigned from your catchment area? What reasons did they give for resigning? - Is resignation in this case a serious matter for management to worry about? | **Supervision - CHWS** |
| 1. **What do you know about sputum submissions? Can you please explain how it is done?** (*Probe more on the process, who collects the sputum, who submits to the facility and how the results reaches the client*)  - What is your monthly target for TB sputum submissions? (*Observe if they know that each SCHW is expected to submit 4 samples every month*) - How many of you have consistently managed to submit 4 samples or above every month? If no, could you please explain reasons for not managing to submit expected samples per month - What are the main challenges that you are facing as SCHWs when it comes to this activity? - How can we improve our catchment area’s performance? | **TB sputum submission** |
| 1. **Overall, how do you feel about your job?**  - What is the one thing that you enjoy /appreciate about your job? - What is the one thing that you dislike/detest about your job? - What is the one thing that you would like to change about the work that you do? - Is there any compelling story/impact case that you can share that encapsulates the value of the work that you do in your assigned households or community? | **Improvement Plan & Wrap Up** |
